# Point Mutations in the *folP* Gene Partly Explain Sulfonamide Resistance of *Streptococcus mutans*

**DOI:** 10.1155/2013/367021

**Published:** 2013-02-25

**Authors:** W. Buwembo, S. Aery, C. M. Rwenyonyi, G. Swedberg, F. Kironde

**Affiliations:** ^1^Department of Anatomy, Makerere University, P.O. Box 7072, Kampala, Uganda; ^2^Department of Medical Biochemistry and Microbiology, Uppsala University, Husargaten 3, Building D7 Level 3, P.O. Box 582, SE-75123 Uppsala, Sweden; ^3^Department of Dentistry, Makerere University, P.O. Box 7072, Kampala, Uganda; ^4^Department of Biochemistry, Makerere University, P.O. Box 7072, Kampala, Uganda

## Abstract

Cotrimoxazole inhibits dhfr and dhps and reportedly selects for drug resistance in pathogens. Here, *Streptococcus mutans* isolates were obtained from saliva of HIV/AIDS patients taking cotrimoxazole prophylaxis in Uganda. The isolates were tested for resistance to cotrimoxazole and their *folP* DNA (which encodes sulfonamide-targeted enzyme dhps) cloned in pUC19. A set of recombinant plasmids carrying different point mutations in cloned folP were separately transformed into *folP*-deficient *Escherichia coli*. Using sulfonamide-containing media, we assessed the growth of *folP*-deficient bacteria harbouring plasmids with differing *folP* point mutations. Interestingly, cloned *folP* with three mutations (A37V, N172D, R193Q) derived from *Streptococcus mutans* 8 conferred substantial resistance against sulfonamide to *folP*-deficient bacteria. Indeed, change of any of the three residues (A37V, N172D, and R193Q) in plasmid-encoded *folP* diminished the bacterial resistance to sulfonamide while removal of all three mutations abolished the resistance. In contrast, plasmids carrying four other mutations (A46V, E80K, Q122H, and S146G) in *folP* did not similarly confer any sulfonamide resistance to *folP*-knockout bacteria. Nevertheless, sulfonamide resistance (MIC = 50 **μ**M) of *folP*-knockout bacteria transformed with plasmid-encoded *folP* was much less than the resistance (MIC = 4 mM) expressed by chromosomally-encoded *folP*. Therefore, *folP* point mutations only partially explain bacterial resistance to sulfonamide.

## 1. Introduction 

*Streptococcus mutans *are commensal bacteria found in the oral cavity [1]. These bacteria which belong to the Viridans Streptococci Group (VSG) cause dental caries and infrequently give rise to extra oral infections like subacute bacterial endocarditis [1, 2]. Although dental caries is not usually treated by antibiotics, the VSG have attracted interest due to their potential to act as reservoirs of resistance to antibiotic determinants [3]. Additionally, in individuals taking antibiotics as prophylaxis, resistance of commensals to antibiotic determinants could be selected [4] and transferred to pathogenic organisms [5] such as *Streptococcus pneumoniae* which kills over 1,000,000 children worldwide every year [4]. 

Cotrimoxazole (SXT) is a combination drug (sulfamethoxazole plus trimethoprim) that is commonly used as prophylaxis in HIV/AIDS patients [6]. Sulfamethoxazole is a long-acting sulphonamide. In addition to wide usage as prophylaxis, SXT is also a highly prescribed drug especially in Sub-Saharan Africa due to its low cost and easy availability. Sub-Saharan Africa is reputed for high antibiotic abuse [7]. In Uganda, SXT is not only highly prescribed in dental practice [8], but also selected for multiple antibiotic resistance in *Streptococcus mutans* among HIV/AIDS patients [7]. Despite these findings, data on the mechanisms of SXT resistance in commensal bacteria such as *Streptococcus mutans* is still scanty. In order to better understand the mechanism of *Streptococcus mutans* resistance to SXT, we characterised the *S. mutans folP* gene that encodes dihydropteroate synthase, the target enzyme of sulfonamides [9]. Previously, we reported [7] that *folP* gene from the highly sulfonamide resistant *S. mutans* isolate 797 did not confer sulfonamide resistance to *E. coli folP* knockout bacteria and that sequencing of the *folA* gene of trimethoprim (TMP) resistant isolates did not reveal any mutations. However, the *folP* gene is very polymorphic [7], and at least one of the variants of *folP* confers high sulfonamide resistance to *E. coli folP* knockout cells. In the current study, we report site-directed mutagenesis experiments in which we altered point mutations in *S*.* mutans folP* gene, inserting the mutagenized *folP* DNA in pUC19 plasmids, which were then transformed into *folP* deficient *E. coli* C600 cells. By assessing the growth of the transformant *E. coli* Delta *folP* cells on media containing different levels of sulfamethoxazole, the influence of individual amino acids on sulfonamide resistance in the *folP *gene knockout bacteria was determined. 

## 2. Methods

**Figure 1:**
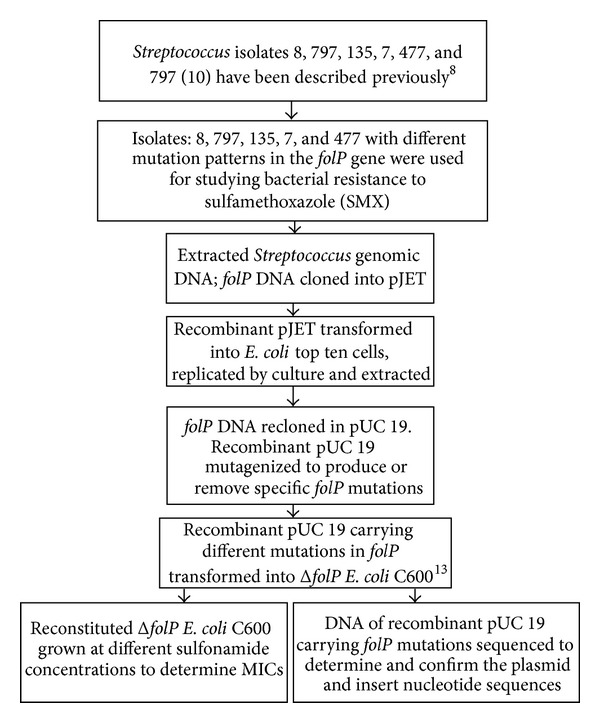
Flow chart showing characterization of *folP* gene. Plasmids carrying *folP* gene were transformed in *folP* gene knockout bacteria to determine the effect of different mutations in plasmid encoded *folP* on bacterial resistance to sulfonamides.

The mechanism of resistance to sulfamethoxazole (SMX) in *S. mutans* was characterised as summarized in Figure 1.

### 2.1. Bacterial Strains and Plasmids

The bacterial strains used in this study (Table 1) were previously isolated [7] from oral specimens of HIV/AIDS patients taking cotrimoxazole as prophylaxis in Kampala, Uganda. The cloning vectors pJet1.2/blunt (Fermentas, Lithuania) and pUC19 [12] were used. *E. coli* top ten cells (Invitrogen, USA) and *E. coli* recipient strain C600 Δ*folP* [13] which is a *folP* knockout strain were used in the transformation experiments. *Streptococcus pneumoniae* ATCC 49619 was used as a susceptible control when determining MICs.

### 2.2. Susceptibility Testing

Minimal inhibitory concentrations (MICs) were determined by the *E*-test method (AB Biodisk, Sweden) following the manufacturer's recommendations. All tests were done on Iso-Sensitest Agar (ISA, Oxoid, UK). Plates were incubated at 37°C in 5% CO_2_ for 24 h. For determination of sulfonamide resistance conferred by cloned *folP* genes, *E. coli* C600 bacteria were grown in Iso-Sensitest Broth (ISB, Oxoid, UK) to a cell density of 10^8^/mL, diluted to 10^4^/mL, and plated on ISA plates containing varying concentrations of sulfathiazole (Sigma Aldrich, USA).

### 2.3. DNA Extraction

Isolates of *Streptococcus mutans* were incubated at 37°C for 12 h on Iso-Sensitest Agar. Bacterial colonies were re-suspended in brain heart infusion broth (BioMérieux, France) and incubated at 37°C for 24 h in an atmosphere of 5% carbon dioxide. Chromosomal bacterial DNA from the cultured broth was then extracted using the Wizard Genomic DNA Purification Kit (Promega, USA). 

### 2.4. Cloning

The PCR primer sequences used were based on the published sequence of the *folP* gene of *Streptococcus mutans* UA159 [10] (Table 1). *FolP *gene amplification was performed in 50-*μ*L volumes containing 0.5 *μ*M of each primer, 100 *μ*M of the four deoxyribonucleoside triphosphates, 5 units of DNA polymerase (Pfu, Fermentas), 2 *μ*L of template DNA (50–500 ng) preparation, and 1X reaction buffer (Pfu, Fermentas) containing 2 mM MgSO_4_. Amplification reactions were performed with the *Eppendorf* mastercycler gradient thermocycler (Eppendorf, Germany) using the following program: heating at 94°C for 2 min, followed by 25 cycles consisting of a denaturation at 94°C for 30 s, annealing at 50°C for 30 s, and an extension at 72°C for 1.5 min. This was followed by a final extension of 72°C for 5 min and a holding step at 16°C. 

The PCR products were cleaned by the PCR Cleanup kit (Omega, USA) and then used for cloning into the pJet vector using the blunt end pJET cloning kit protocol (Fermentas, Lithuania). The ligated products were introduced into *E. coli* top ten cells by heat-shock CaCl_2_ transformation method. 

 Plasmids were prepared from the transformants using the plasmid preparation kit (Omega, USA) and prepared for sequencing.

### 2.5. Sequence Analysis

For sequencing the plasmids, the BigDye Terminator labelled cycle-sequencing kit (Applied Biosystems) and an ABI prism 310 Genetic Analyzer (Applied Biosystems) were used. The results of the *folP* gene sequence analysis were compared with database sequences of *Streptococcus mutans* UA159 [10] and NN2025 [11] using the BLAST programme at NCBI [14]. 

### 2.6. Site-Directed Mutagenesis

Mutagenesis was carried out using a 50 *μ*L reaction mix (Fermentas, Lithuania) containing 1X Pfu buffer with MgSO_4_, 2.5 units Pfu DNA Polymerase (Fermentas, Lithuania), 0.1 mM dNTPs, 10–100 ng of template DNA inserted in pUC19, and 1 *μ*M of each primer (Table 2). The PCR program started with a heating step at 95°C for 30 s, followed by 18 cycles consisting of a denaturation step of 95°C for 30 s, annealing of 50°C for 30 s, and an extension of 68°C for 7 min.

Site-directed mutagenesis products (17 *μ*L of the PCR product) were digested with 5 units of the restriction enzyme Dpn1 (Fermentas, Lithuania) in Buffer Tango to remove unchanged DNA. The mixture was incubated at 37°C for 4 h before incubating at 80°C for 20 min to inactivate Dpn1. 

### 2.7. Transformation

Ten *μ*L of the digested mutagenesis product were transformed into CaCl_2_-treated *folP *knockout *E. coli* competent cells. Recombinant plasmids were prepared from part of a single transformant bacterial colony using the plasmid miniprep kit (Omega, USA). The plasmids were then sequenced as described above to confirm that site-directed mutagenesis had occurred. Bacteria from the same transformant colony were then tested for growth at different SMX concentrations.

## 3. Results 

**Figure 2:**
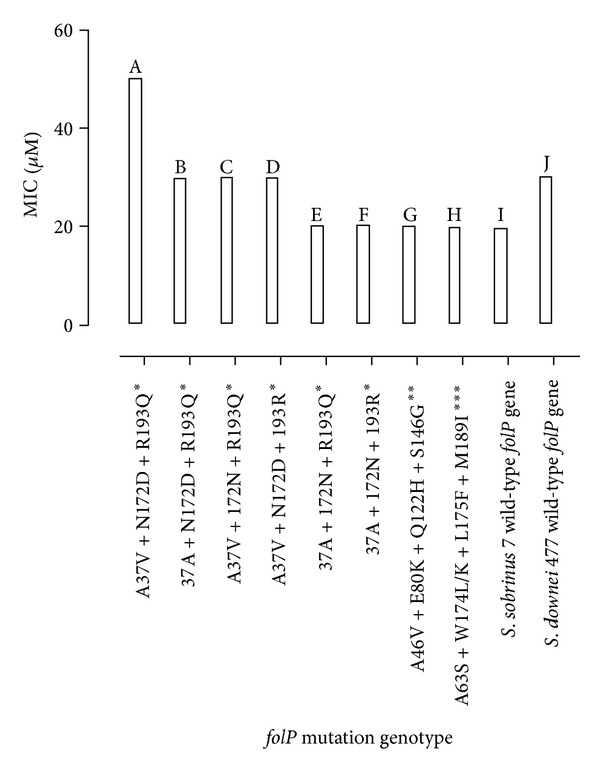
Comparing sulfamethoxazole minimal inhibitory concentrations (MICs) for *folP* knockout *E. coli* cells transformed with pUC19 plasmid carrying differing chromosomal *folP* genes of streptococci. To determine the effect plasmid encoded mutant* folP *has on the sulfamethoxazole resistance of transformed *C600ΔfolP E. coli* bacteria, growths (in SMX containing media) were compared for *folP*-deficient *E. coli* transformed with pUC19 carrying triple-mutant *folP* (residues 37, 172, and 193: bar A), double-mutant *folP* (bars B, C, and D), single-mutant *folP* (residue 193: bar E), and wild-type *folP* (bar F) from *S. mutans* isolate 8. The sulfamethoxazole resistance of transformed *folP* deficient cells was notably increased by transformation with plasmid encoding triple-mutated *folP* (wildtype *folP* MIC = 20 *μ*M, triple-mutant *folP* MIC = 50 *μ*M). Note: the MIC (sulfamethoxazole) for chromosome-encoded *folP* in *S. mutans* isolate 8 was 4 mM (see Table 1). Controls comprising *C600ΔfolP E. coli* transformed with pUC19 encoding either mutant *folP* from *S. mutans* isolates 797 (bar G) and 135 (bar H) or wild-type *folP* from *S. sobrinus* isolate 7 (bar I) or *S. downei* isolate 477 (bar J) showed basal or less sulfamethoxazole resistance (MICs = 20–30 *μ*M). *: *S. mutans* isolate 8 mutant *folP*; **: *S. mutans* isolate 797 mutant *folP*; ***: *S. mutans* isolate 135 mutant *folP*.

The results of transformation of *S. mutans folP* gene into *folP* gene knockout *E. coli* are shown in Figure 2. As previously reported [7], *S. mutans* isolate 797 carries four point mutations (A46V, E80K, Q122H, and S146G) in *folP* gene, as compared to the control strain NN2025 [11] but harbours one such mutation (S146G) if compared to control strain UA159 [12] (Table 1). Two other isolates with DHPS sequence differing from both reference strains were cloned using the same conditions as for 797. These were isolate 8 (A37V, N172D, and R193Q) and isolate 135 (A63S, W174L, L175F, and M189I). Of these, only the cloned *folP* gene from isolate 8 conferred sulfonamide resistance to the *E. coli* C600Δ*folP* recipient. In the present paper, the above-mentioned four point mutations (A46V, E80K, Q122H, and S146G), and those in *S. mutans* isolate 8 (A37V, N172D, and R193Q) (Table 1), were successfully altered or removed from *folP* gene by site-directed mutagenesis. Nonmutagenized chromosomal DNA from isolates *S. sobrinus* 7 and *S. downei* 477 was individually inserted into pUC19 as well. Effects of the different *folP* gene constructs on resistance to sulphonamide were then investigated by transforming the recombinant pUC 19 plasmids in *folP* knockout *E. coli *C600 and assessing the growth of transformant bacteria on media containing different concentrations of sulfamethoxazole (see Figure 1). Interestingly, plasmids harbouring triple mutant *folP *of *S. mutans* isolate 8 (bearing the mutations A37V, N172D, and R193Q) conferred (to *folP* knockout *E. coli*) intermediate level resistance (MIC: 50 *μ*M) against sulfonamide (bar A in Figure 2) even though this is not equal to 4 mM, the MIC arising from the chromosomal *folP* gene in the natural isolate *S. mutans* 8 (Table 1). In addition, altering any of the three polymorphic amino acids (A37V, N172D, and R193Q) back to the UA159 sequence and transforming the double mutant *folP* gene into the *E. coli* Delta-*folP* cells produced reconstituted knockout *E. coli* cells of lower level (MIC: 30 *μ*M) resistance to sulphonamide (Figure 2: bars B, C, D), while reversing two amino acid mutations (Figure 2 bar E) or all three amino acid mutations (Figure 2 bar F) to wild-type and transforming *folP* knockout *E. coli* likewise yielded transformant clones with low resistance to sulphonamide (MIC: 20 *μ*M). On the other hand, as previously reported [7], cloned *folP* gene from 797 did not confer resistance of *folP* deficient *E. coli* C600 to sulphonamide. Moreover, changing the DNA encoding four amino acid mutations (A46V, E80K, Q122H, and S146G) of *folP* in *S. mutans* isolate 797 and transforming the mutant DNA in *folP* knockout *E. coli *to comply with either NN2025 or UA159 sequences (Figure 2 bar G) did not change susceptibility of *folP* knockout bacteria to sulphonamide. Controls consisting of *folP* knockout *E. coli* transformed with pUC 19 plasmids encoding mutant *folP* from *S. mutans *isolate 135 (Figure 2, bar H), wild-type *folP* from *S. sobrinus* 7 (Figure 2 bar I), or wild-type *folP* from *S. downei* 477 produced minimal or reduced resistance. 

## 4. Discussion 

In the present study, we examined Ugandan *Streptococcus mutans* isolates from HIV/AIDS patients who were taking cotrimoxazole as prophylaxis [7]. The isolates were found to have different point mutations in the *folP* gene in relation to wild-type sequences found in databases. It should be noted that these same isolates lacked any mutations in the *folA* gene. 

Resistance to sulfonamides in gram positive bacteria has been shown to be due to mutations in the *folP* gene that render the encoded dihydropteroate synthase insensitive to the drug [15]. In the corresponding gene of *Plasmodium*, more point mutation combinations were previously found to be associated with higher resistance rates against sulfadoxine in *P. falciparum* [16]. In the present study, we assessed the influence of different combinations of mutations in *S. mutans folP* by performing mutagenesis experiments to remove mutations in the *folP* gene and transforming the changed DNA into *folP* knockout *E. coli* cells which were subsequently tested for growth in the presence of varying levels of sulfamethoxazole. We found that the cloned *folP* gene from isolate 8 conferred substantial sulphonamide resistance to *folP* knockout *E. coli* (MIC: 50 *μ*M) (Figure 2) but not to the level observed for the natural isolate *S. mutans* 8 (MIC: 4 mM). Changes in any of the three divergent amino acids (residues 37, 172, and 193) of *folP* reduced the level of resistance to sulfonamide and the removal of all three polymorphisms totally abolished the resistance. In contrast, no combination of the mutations in *folP* from isolate 797 (A46V, E80K, Q122H, and S146G) led to differences in susceptibility of *folP* knockout bacteria to sulfonamide. In addition, we found that isolates 135, 7, and 477 with different mutation patterns in *folP* grew to the same resistance level (MIC: 20 *μ*M) as isolate 797. This finding corroborates the previous report [7] that the cloned *folP* gene from 797 does not confer sulfonamide resistance to *folP*-gene knockout *E. coli* cells. However, that the cloned *folP* gene from isolate *S mutans* 8 did not confer equally high sulphonamide resistance to *folP* knockout *E. coli* as shown by the natural isolate *S. mutans* 8 (MIC: 4 mM) suggests that there is another mechanism of resistance to sulfonamide other than the point mutations. One possibility may be that DHPS synthesis and expression are increased as was found in *Streptococcus agalactiae* [17] or that there may be point mutations in other folate pathway genes. We could not rule out other causes of resistance to sulfonamide in *Streptococcus mutans *since sequencing the *folA* gene of *S.mutans* 8 and flanking regions including the promoter did not reveal any mutations (results not shown). Further experiments including whole genome sequencing of *S. mutans* 8 and other sulfonamide resistant strains may enhance the understanding of sulfa resistance in Streptococci. 

## 5. Conclusions 

Point mutations are one of the explanations for the mechanism of resistance to sulfonamide in *Streptococcus mutans. *However, cloned *folP *gene did not confer full resistance to *folP* knockout cells compared to the original isolate *797*, a result which does not rule out other possible mechanisms for the resistance to sulfonamides. 

## Figures and Tables

**Table 1:** Characteristics of bacterial isolates used in the current study, the respective genes and susceptibility to Cotrimoxazole (STX), sulfamethoxazole (SMX), and trimethoprim (TMP) as determined by *E*-test.

Isolate	Accession number of *folP* gene nucleotide sequence used	Mutations in the *folP* gene	STX	Sulfonamide susceptibility	Trimethoprim susceptibility	Mutations in the *folA* gene
*S. mutans* 8	Not yet submitted to gene banks but previously published [7]	A37V, N172D, and R193Q*	>32 *μ*g/mL	>1024 *μ*g/mL (>4 mM)	>32 *μ*g/mL	None
*S. mutans* 797	HE599533.1	A46V, E80K, Q122H, and S146G**	0.5 *μ*g/mL	>1024 *μ*g/mL (>4 mM)	2 *μ*g/mL	None
*S. mutans* 135	Not yet submitted but previously published [7]	A63S, W174LK, L175F, and M189I**	8 *μ*g/mL	>1024 *μ*g/mL (>4 mM)	0.38 *μ*g/mL	None
*S. sobrinus* 7	HE 599535.1	None	>32 *μ*g/mL	>1024 *μ*g/mL (>4 mM)	>32 *μ*g/mL	None
*S. downei* 477	Similar to ZP 07725257.1	None	0.125 *μ*g/mL	Not done	Not done	Not done

*Mutations as compared to UA159 [10]. **Mutations as compared to NN2025 [11].

**Table 2:** Primers used for cloning and site-directed mutagenesis.

Primer name	Nucleotide sequence

Mutans DHPSph	5′-GAT CGA TCG CAT GCA CAT CAT AAC TAG GGA GCA AGC-3′
mutansDHPSBam	5′-GAT GGA TCG GAT CCA AAA TAATCT TAT CCA TAA CAC CCT CA-3′
dhpssfwph	5′-AAC CTA CTG CAT GCA TAA GAA TCA G-3′
dhpssreveco	5′-ATT GTA GGA ATT CTT CTA GAA AGA TCC-3′
downeifolpfw	5′-GCA TGC CAA AGA CAG GAA TTG CTG AC-3′
Downeifolprevps	5′-CTG CAG CCA CAA AAA TTT GCC CCA GAC-3′

	Primers for changing specific amino acids in isolate 797

DHPS46AVfw	5′-TGA AGC CAT GTT AGT AGC AGG AGC GGC TA-3′
DHPS46AVrev	5′-TAG CCG CTC CTG CTA CTA ACA TGG CTT CA-3′
DHPS80aEKfw	5′-TCG TTC CAA TTG TTA AAG CTA TTA GCG AA-3′
DHPS80aEKrev	5′-TTC GCT AAT AGC TTT AAC AAT TGG AAC GA-3′
DHPS122QHfw	5′-CTT TAT GAT GGG CAC ATG TTT CAA TTA GC-3′
DHPS122QHrev	5′-GCT AAT TGA AAC ATG TGC CCA TCA TAA AG-3′
DHPS146SGfw	5′-GTG AAG AAG TTT ATG GCA ATG TAA CAG AA-3′
DHPS146SGrev	5′-TTC TGT TAC ATT GCC ATA AAC TTC TTC AC-3′

	Primers for changing specific amino acids in isolate 8

V37Afw	5′-AAC CAA TCG ATC AGG CTC TAA AAC AGG TTG A-3′
V37Arev	5′-TCA ACC TGT TTT AGA GCC TGA TCG ATT GTT T-3′
D172Nfw	5′-GGA GTT AAA AAA GAA AAT ATT TGG CTT GAT C-3
D172Nrev	5′-GAT CAA GCC AAA TAT TTT CTT TTT TAA CTC C-3′
Q193Rfw	5′-ACA TGG AAC TTC TAC GAG GCT TAG CGG AGG T-3
Q193Rrev	5′-ACC TCC GCT AAG CCT CGT AGA AGT TCC ATG T-3′
